# Acute abdominal compartment syndrome as a complication of Holmium laser enucleation of the prostate: a case report

**DOI:** 10.1186/1471-2253-14-32

**Published:** 2014-05-12

**Authors:** Torsten Richter, Matthias Huebler

**Affiliations:** 1 Department of Anesthesia and Intensive Care, Carl Gustav Carus University Hospital, TU Dresden, Fetscherstrasse 74, 01307 Dresden, Germany

**Keywords:** Abdominal compartment, Intra-abdominal hypertension, Transurethral resection of prostate

## Abstract

**Background:**

In 1996, Holmium laser enucleation of the prostate was introduced and has been shown to be safe and highly effective.

**Case presentation:**

We report a case of a rare complication that resulted in intra-abdominal compartment syndrome with prolonged intubation and intensive care, involving an 74-year-old male after holmium laser enucleation of prostate, with a massive irrigant fluid leakage into the retroperitoneal space. The elevated abdominal pressure was reduced by forced diuresis. The tracheal tube was removed 18 hours after the patient’s transfer to the ICU. The patient was discharged to home one week after the operation.

**Conclusion:**

In rare cases when no obvious ruptures of the prostate capsule or the bladder occur during laser enucleation of prostate, knowledge regarding possible emersion of massive amounts of irrigant fluid into the retroperitoneal space leading to intra-abdominal compartment syndrome aids in the diagnosis and subsequent successful therapy of intra-abdominal hypertension.

## Background

Transurethral resection of the prostate (TURP) has been developed over the years and can be performed using different techniques. Holmium laser enucleation of the prostate (HoLEP) allows resection of large prostatic hyperplasias. During the procedure, the laser fibre dissects the prostatic lobes off the surgical capsule similar to how a surgeon’s finger would during an open, transvesicular prostatectomy. First, the enucleated lobes are pushed back into the bladder. At the end of the procedure, the large prostatic tissue chunks are reduced to small pieces using a soft tissue morcellator to allow evacuation from the bladder [1]. The HoLEP technique was introduced in 1996 and has been shown to be safe and highly effective [2]. One rare complication is capsular perforation [3], but no case of intra-abdominal compartment syndrome has been reported thus far. Here, we present a case of massive irrigant fluid leakage into the retroperitoneal space leading to intestinal paralysis and intra-abdominal hypertension.

## Case presentation

A 74-year-old patient was scheduled for HoLEP to treat his prostatic hyperplasia (estimated prostatic volume 80 ml). His weight and height were 85 kg and 166 cm, respectively. He had arterial hypertension, hypothyreosis, hyperlipidaemia and coronary artery disease with confirmed stenosis of one coronary vessel. On the day of surgery, he received his usual medications (candesartan cilexetil 16 mg, metoprolol succinate 95 mg, levothyroxine 75 μg, amlodipine 5 mg, and ciprofloxacine 250 mg) orally. He was premedicated 30 min before the operation with 7.5 mg midazolam (Roche Pharma AG, Grenzach-Whylen, Germany) by mouth. A peripheral venous access catheter was placed, and standard monitoring (ECG, blood pressure, and pulse oximetry) was established. General anaesthesia was induced using 2 mg/kg propofol (Fresenius Kabi, Bad Homburg, Germany) and 300 μg fentanyl (Hameln pharma plus GmbH, Hameln, Germany). Uneventful tracheal intubation was facilitated using 50 mg atracurium (Hameln pharmaceuticals GmbH, Hameln, Germany). Anaesthesia was maintained using repetitive administrations of fentanyl and 0.9-1.0 MAC of desflurane (Baxter, Unterschleissheim, Germany). The patient was ventilated in a volume-controlled mode with the following ventilator settings: tidal volume 440 ml, PEEP 6 cm H_2_O, respiratory rate 12/min, ratio inspiration:expiration (I:E) 1:1.7. Peak inspiratory pressure (P_peak_) was 24 cm H_2_O. The patient was placed in the lithotomy position for the surgical procedure.

**Figure 1:**
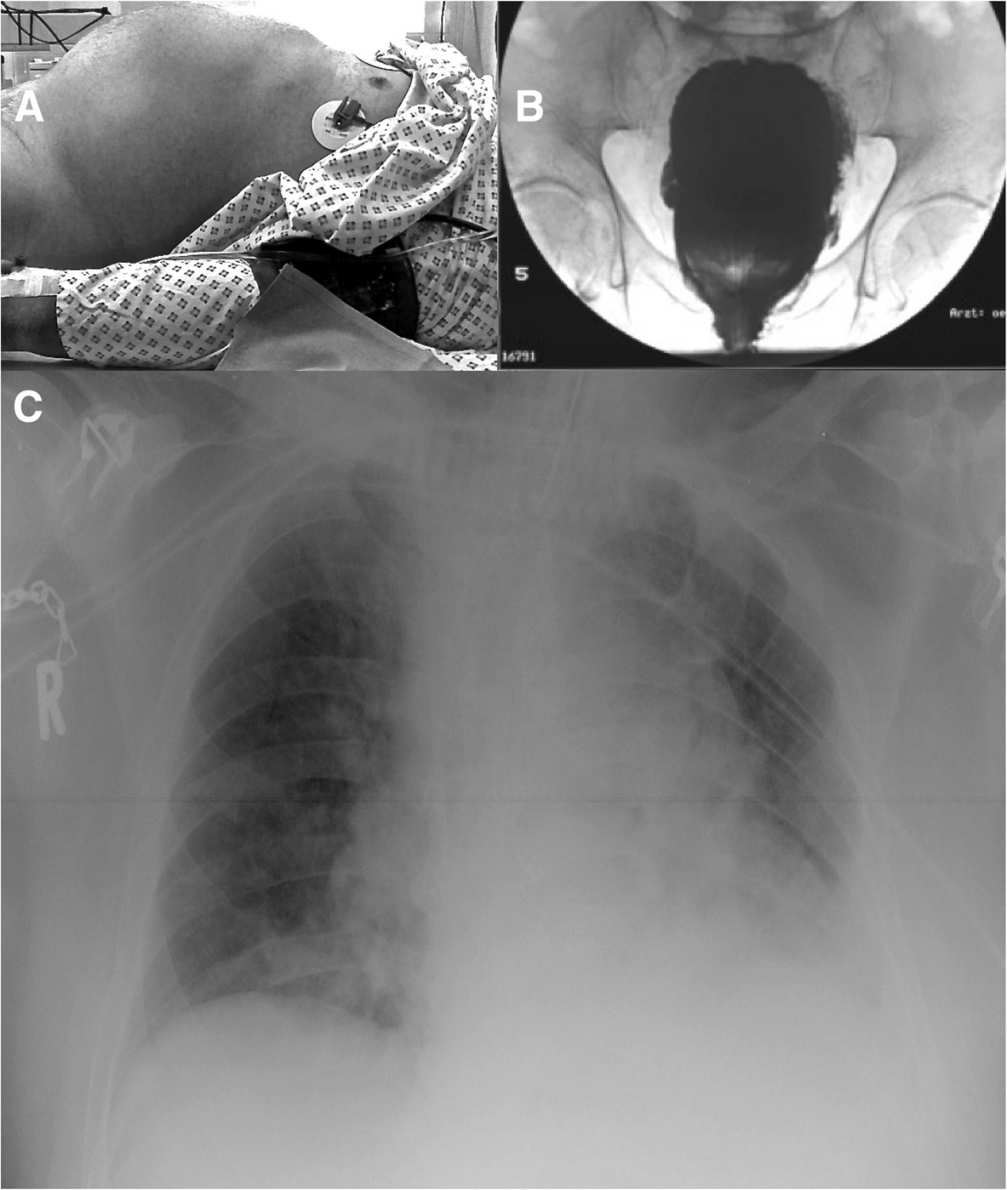
**Patient images. A)** Patient on the x-ray table. Please note the prominent abdominal region. Measured intra-abdominal pressure was 26 mmHg. **B)** Contrast agent x-ray of the bladder. There are no signs of bladder perforation. **C)** X-ray of the patient’s thorax during ventilation. Endotracheal tube and central venous line were in the correct position. There were signs of atelectasis of the left lower lung and cephalic displacement of the diaphragm due to the intra-abdominal volume augmentation and despite the restricted conditions for the x-ray in the ICU bed.

One hour after the start of the surgery, P_peak_ increased to 34 cm H_2_O. All other cardiovascular and respiratory parameters remained unchanged. The ventilator settings and the position and integrity of the tracheal tube were checked. The I:E was changed to 1:1, and 300 μg fentanyl was administered without affecting the P_peak_. The surgeon noted a deterioration in surgical conditions due to increased tension. The surgery lasted 3.5 hours due to technical problems with the laser. P_peak_ increased continuously and was 39 cm H_2_O at the end of the procedure. During the final morcellation of the prostatic tissue, a very tender abdomen was noted and vesicular perforation with intra-abdominal irrigant fluid extravasation were suspected. Abdominal ultrasound showed free peri-hepatic fluid. The patient was transferred to the x-ray unit to undergo contrast agent imaging studies. An intra-abdominal bladder perforation was excluded (Figure 1); however, suspicion of a perforation of the prostatic capsule with access to the retroperitoneal space remained. The patient received a single dose of diuretics (40 mg furosemide) intravenously.

The change in the patient’s position from lithotomy to supine induced a drop in blood pressure, which required continuous administration of norepinephrine (0.2-0.4 mg/h). A central venous line and invasive blood pressure monitoring were established. Chest x-ray showed an elevated diaphragm and consolidation of lung tissue, which was indicative of atelectasis (Figure 1). There were no signs of acute pulmonary venous congestion. We decided to transfer the patient to the ICU for prolonged weaning.

The intravesical pressure was 26 mmHg and confirmed the suspected intra-abdominal compartment syndrome. The urine output was stable (100–400 ml/h) after ICU admission and normalised without further treatment with diuretics. The fluid balance was -1500 ml within 24 hours. The bladder pressure dropped to normal values (12 mmHg) 14 hours after ICU admission. The tracheal tube was removed 18 hours after the patient’s transfer to the ICU. The abdomen was still distended but soft. The remainder of the postoperative course was uneventful, and the patient was discharged to home on the 7th day after the operation.

## Discussion

Transurethral prostatic resections are very common procedures, and the most frequent complication, which is of concern for the anaesthesiologist, is TUR syndrome due to hypotonic hyperhydration. One advantage of the HoLEP technique is that an isotonic irrigant solution is used and TUR syndrome is impossible. During the procedure, the irrigant solution is allowed to flow continuously without intermittent vesicular pressure relief. Typically, the irrigation bag is only lifted 50–80 cm above the patient’s level. The mean surgical time is longer compared to TURP and the number of catheterisations, the length of hospital stay and the risk of adverse events are lower [4]. Typical complications include bleeding, capsular perforation, superficial bladder mucosal injury and bladder perforation, which can occur during different phases of the procedure [3]. The coagulative properties of the holmium laser seal the vessels and prevent absorption of the irrigant.

In our patient, we suspected bladder perforation with intra-abdominal irrigant fluid to be responsible for the intra-abdominal compartment syndrome. However, the x–ray exam found an intact bladder wall with some leakage of contrast agent at the site of the prostatic capsule. This perforation had occurred during the HoLEP procedure without the surgeon’s notice. Symptomatic therapy with diuretics and respiratory support has been shown to be effective in these cases, and retropubic drainage has been mentioned in a case of extensive extravasation [5]. Ultrasound is a useful tool for early detection of fluid extravasation. This is also true if retroperitoneal volume shifts are in focus of the exam provided that the investigator has the expertise [6-8].

The size of the prostate gland has not been shown to have a negative impact on the efficiency of HoLEP in the hands of experienced urologists [9]. The rate of complications drops as the number of operations performed by the primary surgeon increases. It has been reported that after 20 cases under supervision, outcomes similar to those of a more experienced surgeons can be expected [10]. However, the unobserved perforation of the prostatic capsule resulted in a significant increase in retroperitoneal fluid with a subsequent increase in abdominal pressure. The resulting abdominal compartment syndrome caused several known problems in this patient, including deterioration of ventilation (caused by cephalic displacement of the diaphragm resulting in increased inspiratory and mean airway pressures, alveolar atelectasis, reduced capillary flow, arterial hypoxemia and hypercarbia), renal dysfunction (manifested as an increase in creatinine and a drop in the glomerular filtration rate), and reduction of perfusion to the abdominal organs (impairment of the gastrointestinal system and the hepatobiliary system). Therefore, we provided supportive treatment in the ICU with extended weaning and blood pressure stabilisation using noradrenaline. The elevated abdominal pressure was initially reduced by forced diuresis (furosemide administration). Supportive continuous catecholamine administration was stopped shortly after extubation the following day.

## Conclusion

If in rare cases, the rupture of prostatic capsule may occured, but undetected, knowledge regarding possible extravasation of massive amounts of irrigant fluid into the retroperitoneal space leading to intra-abdominal compartment syndrome aids in the diagnosis and subsequent successful treatment of intra-abdominal hypertension.

## Consent

A written informed consent was obtained from the patient for publication of this case report.

## Abbreviations

ECG: Electrocardiogram; HoLEP: Holmium laser enucleation of the prostate; ICU: Intensive care unit; I:E: Inspiration:expiration; MAC: Minimum alveolar concentration; Ppeak: Peak inspiratory pressure; PEEP: Positive end-expiratory pressure; TURP: Transurethral resection of the prostate.

## Competing interests

The authors declare that they have no competing interests. Support was provided solely from department sources.

## Authors’ contributions

TR and MH: Preparation of the manuscript and involvement in the case. The authors read and approved the final manuscript.

## Pre-publication history

The pre-publication history for this paper can be accessed here:

http://www.biomedcentral.com/1471-2253/14/32/prepub
